# The genome sequence of the tree of heaven, *Ailanthus altissima* (Mill.) Swingle, 1916

**DOI:** 10.12688/wellcomeopenres.19628.1

**Published:** 2023-07-25

**Authors:** Rowan J. Schley, Ilia J. Leitch, Maarten J. M. Christenhusz, Royal Botanic Gardens Kew Genome Acquisition Lab, Darwin Tree of Life Barcoding collective, Plant Genome Sizing collective, Wellcome Sanger Institute Tree of Life programme, Wellcome Sanger Institute Scientific Operations: DNA Pipelines collective, Tree of Life Core Informatics collective, Darwin Tree of Life Consortium

**Affiliations:** 1 University of Exeter, Exeter, England, UK; 2 Royal Botanic Gardens Kew, Richmond, England, UK

**Keywords:** Ailanthus altissima, tree of heaven, genome sequence, chromosomal, Simaroubaceae

## Abstract

We present a genome assembly from an individual *Ailanthus altissima* (tree of heaven; Streptophyta; Magnoliopsida; Sapindales; Simaroubaceae). The genome sequence is 939 megabases in span. Most of the assembly is scaffolded into 31 chromosomal pseudomolecules. The mitochondrial and plastid genome assemblies are 661.1 kilobases and 161.1 kilobases long, respectively.

## Species taxonomy

Eukaryota; Viridiplantae; Streptophyta; Embryophyta; Tracheophyta; Spermatophyta; Magnoliopsida; eudicotyledons; Gunneridae; Pentapetalae; rosids; malvids; Sapindales; Simaroubaceae; *Ailanthus*; *Ailanthus altissimus* (Mill.) Swingle, 1916 (NCBItxid:23810).

## Background

**Figure 1.**
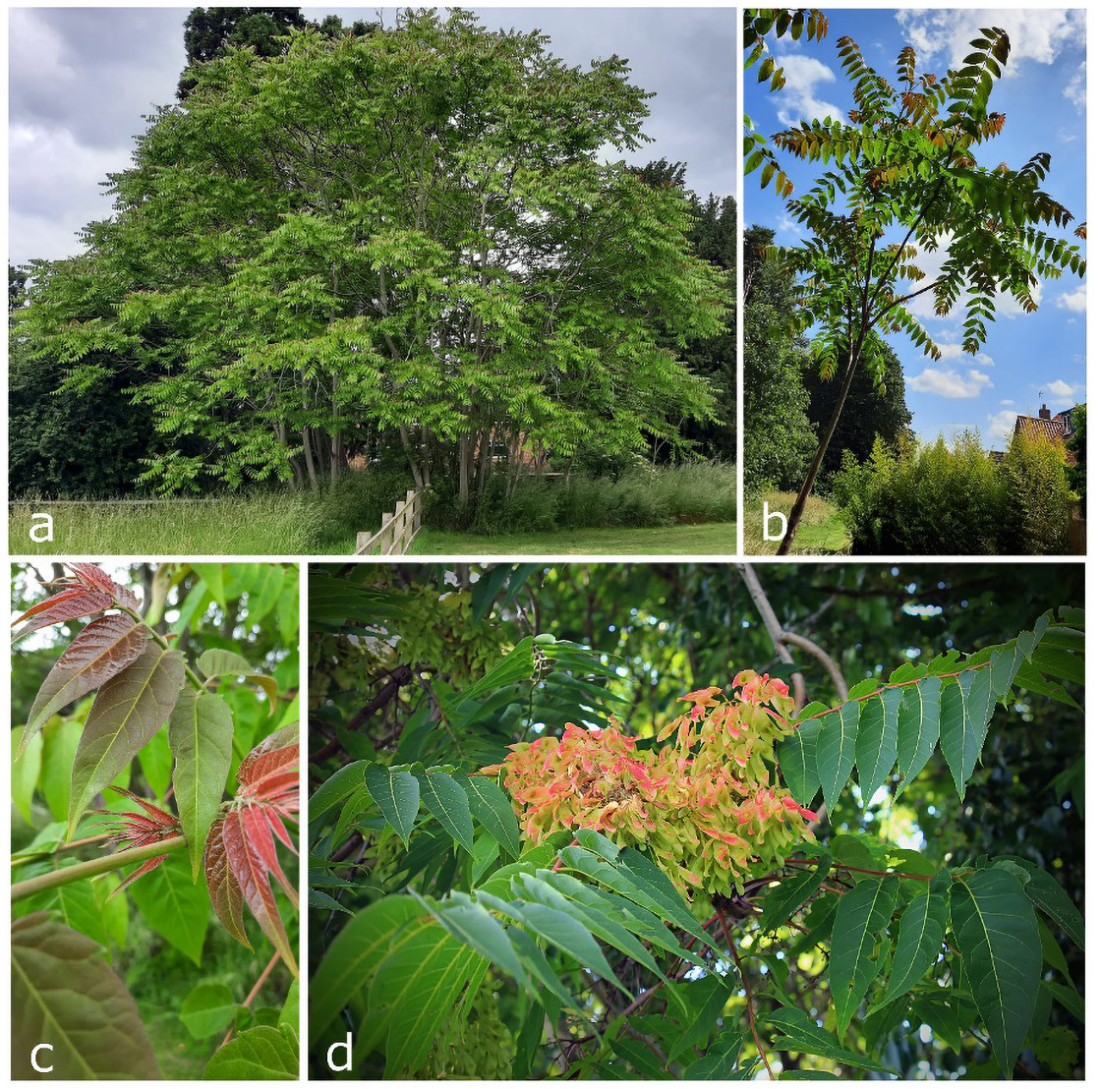
*Ailanthus altissima* (not the sampled specimen). **a**) mature tree; **b**) young sapling, and **c**) new leaves in Nottinghamshire (photographs © Carlton Roberts); **d**) *A. altissima* fruits (photograph © Nicholas *A. Tonelli*).

The tree of heaven ( *Ailanthus altissima*, Simaroubaceae) is a deciduous tree native to China and Taiwan, known for its fast growth and attractive foliage. For these reasons, it has been widely planted as an ornamental tree ( Figure 1). *Ailanthus altissima* was first introduced to the UK in 1751 as a garden plant ( Hu, 1979), during a period when European fascination with Chinese culture (‘ *Chinoiserie*’) was at its height. *Ailanthus altissima* has long been used in Chinese traditional medicine as well as in silk production, as a foodplant for the *Ailanthus* silk moth ( *Samia cynthia*) ( Swingle, 1916). *Ailanthus altissima* has since become an invasive species across North America, southern South America, South Africa, New Zealand and Europe due to its rapid growth, resistance to pollution, fecundity, vegetative reproduction and use of allelopathic chemicals to stifle competition ( Demeter *et al.*, 2021).

The genus name *Ailanthus* is derived from the Ambonese word ‘ailanto’, meaning ‘heaven-tree’, and the specific epithet *altissima* is Latin for ‘tallest’, referring to the size of the mature tree. Three varieties of *A. altissima* have been described – *A. altissima* var. *altissima*, native to mainland China, *A. altissima* var. *sutchuenensis* (Dode) Rehder and E. H. Wilson, restricted to southern China and *A. altissima* var. *tanakae* (Hayata) Kaneh. and Sasaki, endemic to northern Taiwan. Multiple chromosome counts have been reported for *A. altissima*, ranging from 2 *n* = 80 for arboretum specimens examined at the Royal Botanic Gardens, Kew ( Desai, 1960), down to 2 *n* = 64 for samples collected from naturalised populations in mainland Europe ( Ivanova *et al.*, 2006), suggesting polyploidy with different cytotypes between individuals.

Here we present the first high-quality genome of *A. altissima* var. *altissima*, which we believe will be a useful resource both for those studying the tree in its native range and those aiming to understand what underlies its success as an invasive species. This genome complements existing studies which identified microsatellites for use in population genetic analysis ( Dallas *et al.*, 2005; Saina *et al.*, 2021), and those which sequenced the plastome of the species ( Saina *et al.*, 2018). Fruitful directions for future work may include investigating the population genetics of invasion, building on previous work showing population bottlenecks in invasive populations of *A. altissima* ( Neophytou *et al.*, 2020).

## Genome sequence report

The genome was sequenced from the leaves of an *A. altissima* specimen (drAilAlti1) collected from the Royal Botanic Gardens, Kew (latitude 51.482, longitude –0.2879). Using flow cytometry, the genome size (1C-value) was estimated as 1.20 pg, equivalent to 1,170 Mb. A total of 45-fold coverage in Pacific Biosciences single-molecule HiFi long reads and 35-fold coverage in 10X Genomics read clouds were generated. Primary assembly contigs were scaffolded with chromosome conformation Hi-C data. Manual assembly curation corrected 17 missing joins or misjoins and removed one haplotypic duplication, reducing the assembly length by 0.11% and the scaffold number by 33.93%, and decreasing the scaffold N50 by 3.45%.

**Figure 2.**
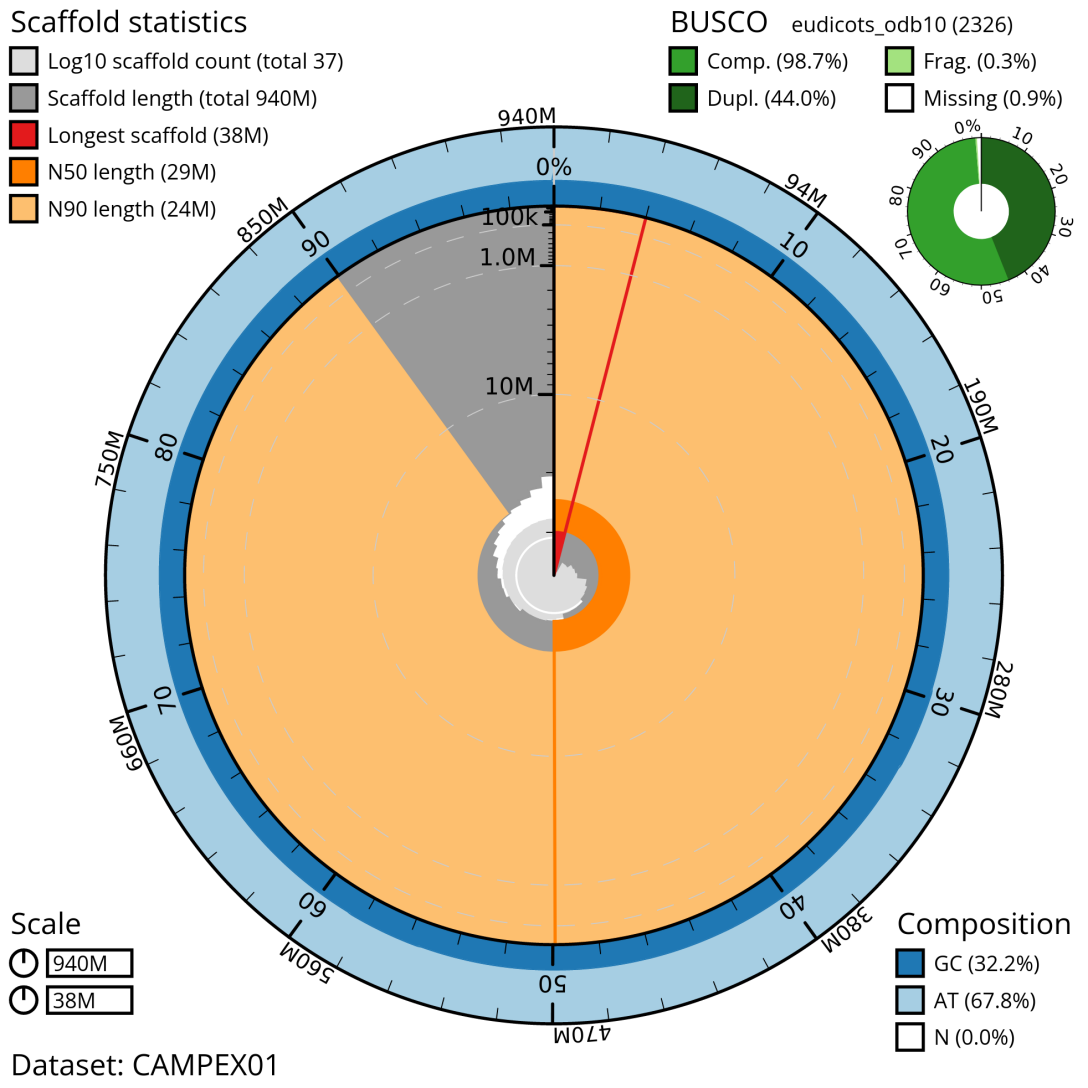
Genome assembly of *Ailanthus altissima,* drAilAlti1.1: metrics. The BlobToolKit Snailplot shows N50 metrics and BUSCO gene completeness. The main plot is divided into 1,000 size-ordered bins around the circumference with each bin representing 0.1% of the 939,218,608 bp assembly. The distribution of scaffold lengths is shown in dark grey with the plot radius scaled to the longest scaffold present in the assembly (38,427,504 bp, shown in red). Orange and pale-orange arcs show the N50 and N90 scaffold lengths (29,421,908 and 24,184,688 bp, respectively). The pale grey spiral shows the cumulative scaffold count on a log scale with white scale lines showing successive orders of magnitude. The blue and pale-blue area around the outside of the plot shows the distribution of GC, AT and N percentages in the same bins as the inner plot. A summary of complete, fragmented, duplicated and missing BUSCO genes in the eudicots_odb10 set is shown in the top right. An interactive version of this figure is available at https://blobtoolkit.genomehubs.org/view/CAMPEX01/dataset/CAMPEX01/snail.

The final assembly has a total length of 939 Mb in 37 sequence scaffolds with a scaffold N50 of 29.4 Mb ( Table 1). Most of the assembly sequence (97.72%) was assigned to 31 chromosomal-level scaffolds ( Figure 2– Figure 5; Table 2). Several small repetitive scaffolds localise equally well to both chromosome 28 and chromosome 9. They have been placed with chromosome 9, although their placement is uncertain. While not fully phased, the assembly deposited is of one haplotype. Contigs corresponding to the second haplotype have also been deposited.

**Table 1.** Genome data for *Ailanthus altissima,* drAilAlti1.1.

Project accession data		
Assembly identifier	drAilAlti1.1	
Species	*Ailanthus altissima*	
Specimen	drAilAlti1	
NCBI taxonomy ID	23810	
BioProject	PRJEB47393	
BioSample ID	SAMEA7522284	
Isolate information	Monoecious; leaves	
Assembly metrics *		*Benchmark*
Consensus quality (QV)	61.1	*≥ 50*
*k*-mer completeness	100%	*≥ 95%*
BUSCO **	C:98.7%[S:54.7%,D:44.0%], F:0.3%,M:0.9%,n:2,326	*C ≥ 95%*
Percentage of assembly mapped to chromosomes	97.72%	*≥ 95%*
Sex chromosomes	Not applicable	*localised homologous pairs*
Organelles	MT single scaffold of 661,054 bp Plastid single scaffold of 161,079 bp	*complete single alleles*
Raw data accessions		
PacificBiosciences SEQUEL II	ERR6808067, ERR6939282	
10X Genomics Illumina	ERR6745740–ERR6745743	
Hi-C Illumina	ERR6745744	
PolyA RNA-Seq Illumina	ERR9435026, ERR9435027	
Genome assembly		
Assembly accession	GCA_946807835.1	
*Accession of alternate haplotype*	GCA_946808065.1	
Span (Mb)	939	
Number of contigs	49	
Contig N50 length (Mb)	29.4 Mb	
Number of scaffolds	37	
Scaffold N50 length (Mb)	29.3 Mb	
Longest scaffold (Mb)	38.4	

* Assembly metric benchmarks are adapted from column VGP-2020 of “Table 1: Proposed standards and metrics for defining genome assembly quality” from ( Rhie *et al.*, 2021). ** BUSCO scores based on the eudicots_odb10 BUSCO set using v5.3.2. C = complete [S = single copy, D = duplicated], F = fragmented, M = missing, n = number of orthologues in comparison. A full set of BUSCO scores is available at https://blobtoolkit.genomehubs.org/view/CAMPEX01/dataset/CAMPEX01/busco.

**Figure 3.**
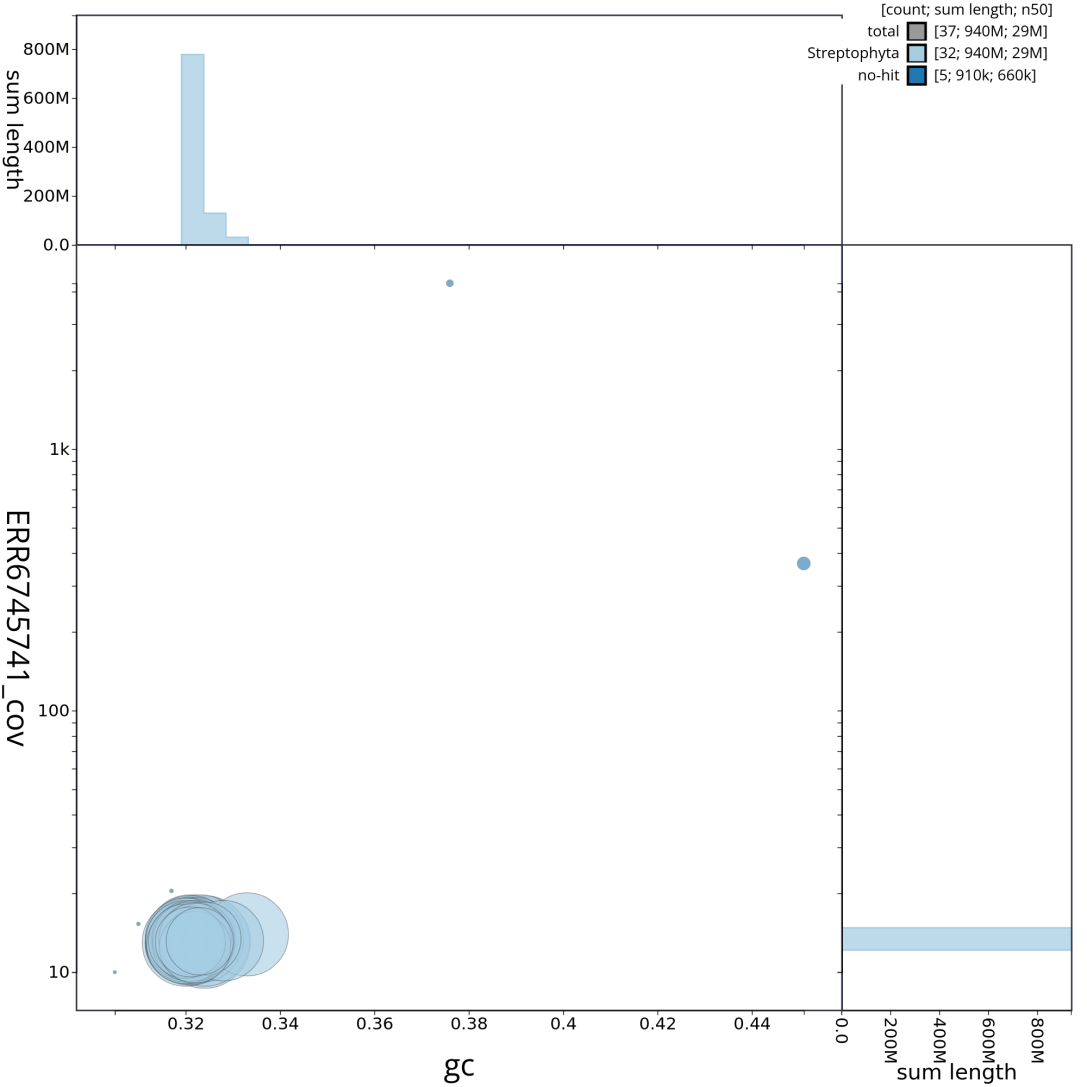
Genome assembly of *Ailanthus altissima,* drAilAlti1.1: GC coverage. BlobToolKit GC-coverage plot. Chromosomes are coloured by phylum. Circles are sized in proportion to chromosome length. Histograms show the distribution of chromosome length sum along each axis. An interactive version of this figure is available at https://blobtoolkit.genomehubs.org/view/CAMPEX01/dataset/CAMPEX01/blob.

**Figure 4.**
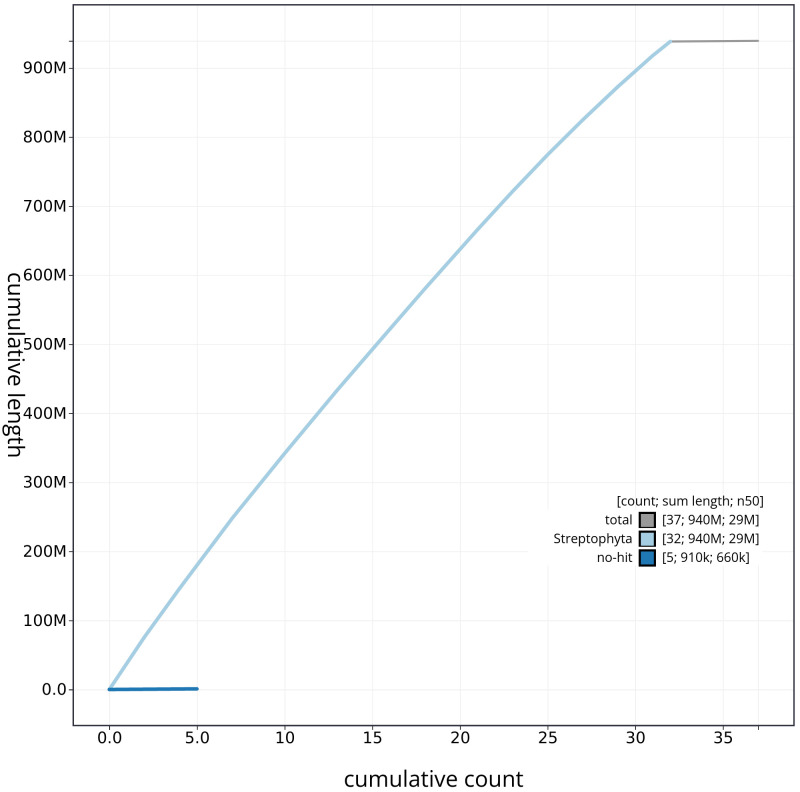
Genome assembly of *Ailanthus altissima,* drAilAlti1.1: cumulative sequence. BlobToolKit cumulative sequence plot. The grey line shows cumulative length for all chromosomes. Coloured lines show cumulative lengths of chromosomes assigned to each phylum using the buscogenes taxrule. An interactive version of this figure is available at https://blobtoolkit.genomehubs.org/view/CAMPEX01/dataset/CAMPEX01/cumulative.

**Figure 5.**
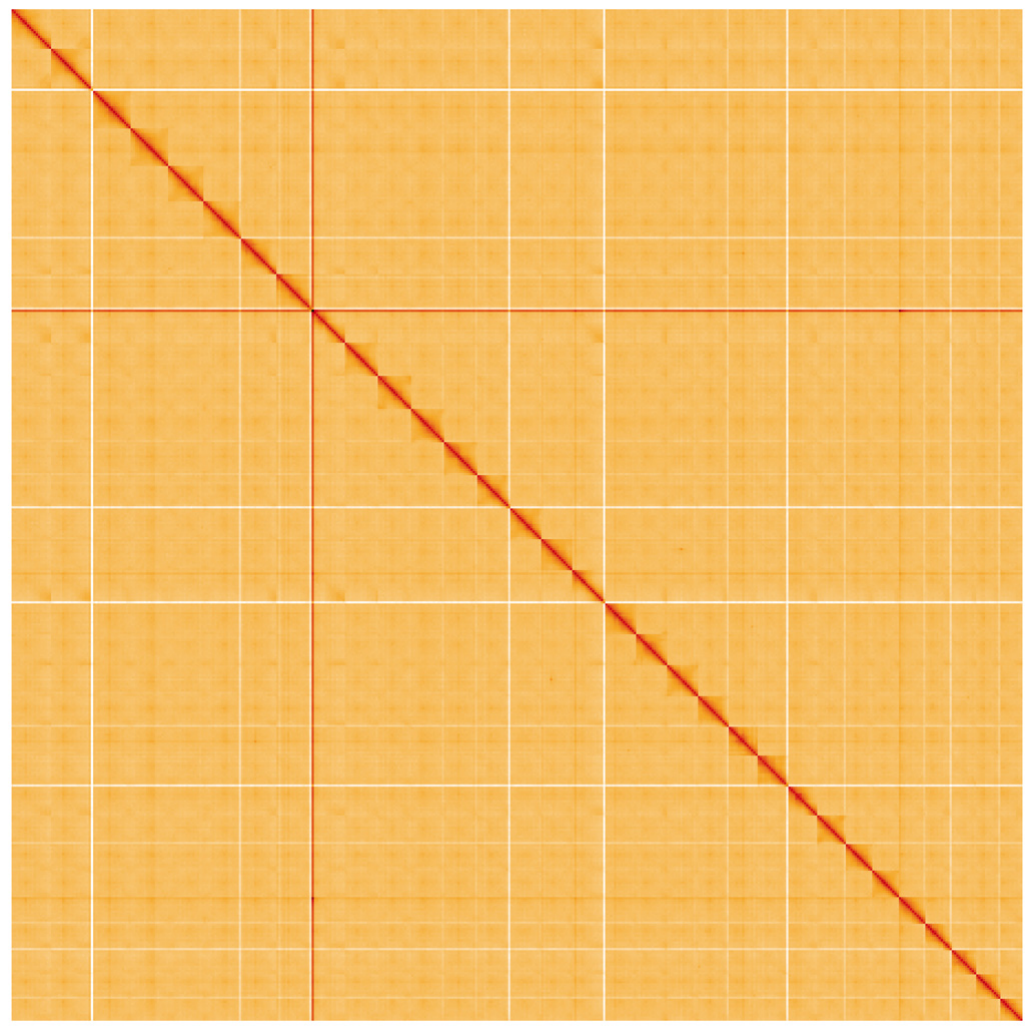
Genome assembly of *Ailanthus altissima,* drAilAlti1.1: Hi-C contact map. Hi-C contact map of the drAilAlti1.1 assembly, visualised using HiGlass. Chromosomes are given in order of size from left to right and top to bottom. An interactive version of this figure is available at https://genome-note-higlass.tol.sanger.ac.uk/l/?d=XyRKDNDOTHy19SzkZkKRWw.

**Table 2.** Chromosomal pseudomolecules in the genome assembly of *Ailanthus altissima*, drAilAlti1.

INSDC accession	Chromosome	Size (Mb)	GC%
OX327683.1	1	38.43	32.4
OX327684.1	2	37.36	32.4
OX327685.1	3	35.13	32.1
OX327686.1	4	35.07	32.3
OX327687.1	5	34.17	32
OX327688.1	6	33.92	32.1
OX327689.1	7	33.54	32.1
OX327690.1	8	31.7	32.1
OX327691.1	9	31.31	33.3
OX327692.1	10	31.18	32.1
OX327693.1	11	30.58	32.1
OX327694.1	12	30.54	32.2
OX327695.1	13	30.47	32.1
OX327696.1	14	29.5	32
OX327697.1	15	29.42	32
OX327698.1	16	29.33	32
OX327699.1	17	29.31	32.8
OX327700.1	18	29.28	32
OX327701.1	19	28.68	32
OX327702.1	20	28.67	32.1
OX327703.1	21	28.14	32.2
OX327704.1	22	27.98	32.2
OX327705.1	23	27.39	32
OX327706.1	24	27.08	32.1
OX327707.1	25	26.09	32.1
OX327708.1	26	25.42	32.1
OX327709.1	27	24.94	32
OX327710.1	28	24.18	32.4
OX327711.1	29	23.78	32.2
OX327712.1	30	23.17	32.2
OX327713.1	31	22.13	32.1
OX327714.1	MT	0.66	45.1
OX327715.1	Pltd	0.16	

The estimated Quality Value (QV) of the final assembly is 61.1 with *k*-mer completeness of 100%, and the assembly has a BUSCO v5.3.2 completeness of 98.7% (single 54.7%, duplicated 44.0%), using the eudicots_odb10 reference set ( *n* = 2,326).

Metadata for specimens, spectral estimates, sequencing runs, contaminants and pre-curation assembly statistics can be found at https://links.tol.sanger.ac.uk/species/23810.

## Methods

### Sample acquisition, genome size estimation and nucleic acid extraction

*Ailanthus altissima* grows as a weed along pavements and in neglected plots in the UK. Leaves from an *A. altissima* individual (drAilAlti1) were harvested from a plant growing in the pavement near the Jodrell Gate, Royal Botanic Gardens Kew, Surrey, UK (latitude 51.482, longitude –0.2879). The sample was collected and identified by Maarten Christenhusz (Royal Botanic Gardens, Kew). The leaves were preserved by freezing at –80°C.

The genome size was estimated by flow cytometry using the fluorochrome propidium iodide and following the ‘one-step’ method as outlined in ( Pellicer *et al.*, 2021). Specifically for this species, the General Purpose Buffer (GPB) supplemented with 3% PVP and 0.08% (v/v) beta-mercaptoethanol was used for isolation of nuclei ( Loureiro *et al.*, 2007), and the internal calibration standard was *Petroselinum crispum* ‘Champion Moss Curled’ with an assumed 1C-value of 2,200 Mb ( Obermayer *et al.*, 2002).

DNA was extracted at the Tree of Life laboratory, Wellcome Sanger Institute (WSI). The drAilAlti1 sample was weighed and dissected on dry ice with tissue set aside for Hi-C sequencing. Leaf tissue was cryogenically disrupted to a fine powder using Qiagen Plant Magattract, receiving multiple impacts. High molecular weight (HMW) DNA was extracted using the Qiagen MagAttract HMW DNA extraction kit. Low molecular weight DNA was removed from a 20 ng aliquot of extracted DNA using 0.8X AMpure XP purification kit prior to 10X Chromium sequencing; a minimum of 50 ng DNA was submitted for 10X sequencing. HMW DNA was sheared into an average fragment size of 12–20 kb in a Megaruptor 3 system with speed setting 30. Sheared DNA was purified by solid-phase reversible immobilisation using AMPure PB beads with a 1.8× ratio of beads to sample to remove the shorter fragments and concentrate the DNA sample. The concentration of the sheared and purified DNA was assessed using a Nanodrop spectrophotometer and Qubit Fluorometer and Qubit dsDNA High Sensitivity Assay kit. Fragment size distribution was evaluated by running the sample on the FemtoPulse system.

RNA was extracted from leaf tissue of drAilAlti1 in the Tree of Life Laboratory at the WSI using TRIzol, according to the manufacturer’s instructions. RNA was then eluted in 50 μL RNAse-free water and its concentration was assessed using a Nanodrop spectrophotometer and Qubit Fluorometer using the Qubit RNA Broad-Range (BR) Assay kit. Analysis of the integrity of the RNA was done using Agilent RNA 6000 Pico Kit and Eukaryotic Total RNA assay.

### Sequencing

Pacific Biosciences HiFi circular consensus and 10X Genomics read cloud DNA sequencing libraries were constructed according to the manufacturers’ instructions. Poly(A) RNA-Seq libraries were constructed using the NEB Ultra II RNA Library Prep kit. DNA and RNA sequencing were performed by the Scientific Operations core at the WSI on Pacific Biosciences SEQUEL II (HiFi), Illumina HiSeq 4000 (RNA-Seq) and Illumina NovaSeq 6000 (10X) instruments. Hi-C data were also generated from material from drAilAlti1 using the Arima v2 kit and sequenced on the Illumina NovaSeq 6000 instrument.

### Genome assembly, curation and evaluation

Assembly was carried out with Hifiasm ( Cheng *et al.*, 2021) and haplotypic duplication was identified and removed with purge_dups ( Guan *et al.*, 2020). One round of polishing was performed by aligning 10X Genomics read data to the assembly with Long Ranger ALIGN, calling variants with FreeBayes ( Garrison & Marth, 2012). The assembly was then scaffolded with Hi-C data ( Rao *et al.*, 2014) using SALSA2 ( Ghurye *et al.*, 2019). The assembly was checked for contamination and corrected using the gEVAL system ( Chow *et al.*, 2016) as described previously ( Howe *et al.*, 2021). Manual curation was performed using gEVAL, HiGlass ( Kerpedjiev *et al.*, 2018) and Pretext ( Harry, 2022). The mitochondrial and plastid genomes were assembled using MBG ( Rautiainen & Marschall, 2021) from PacBio HiFi reads mapping to related genomes. A representative circular sequence was selected for each from the graph based on read coverage.

A Hi-C map for the final assembly was produced using bwa-mem2 ( Vasimuddin *et al.*, 2019) in the Cooler file format ( Abdennur & Mirny, 2020). To assess the assembly metrics, the *k*-mer completeness and QV consensus quality values were calculated in Merqury ( Rhie *et al.*, 2020). This work was done using Nextflow ( Di Tommaso *et al.*, 2017) DSL2 pipelines “sanger-tol/readmapping” ( Surana *et al.*, 2023a) and “sanger-tol/genomenote” ( Surana *et al.*, 2023b). The genome was analysed within the BlobToolKit environment ( Challis *et al.*, 2020) and BUSCO scores ( Manni *et al.*, 2021; Simão *et al.*, 2015) were calculated.

Table 3 contains a list of relevant software tool versions and sources.

**Table 3.** Software tools: versions and sources.

Software tool	Version	Source
BlobToolKit	3.3.8	https://github.com/blobtoolkit/blobtoolkit
BUSCO	5.3.2	https://gitlab.com/ezlab/busco
FreeBayes	1.3.1-17-gaa2ace8	https://github.com/freebayes/freebayes
gEVAL	N/A	https://geval.org.uk/
Hifiasm	0.15.3	https://github.com/chhylp123/hifiasm
HiGlass	1.11.6	https://github.com/higlass/higlass
Long Ranger ALIGN	2.2.2	https://support.10xgenomics.com/genome-exome/ software/pipelines/latest/advanced/other-pipelines
Merqury	MerquryFK	https://github.com/thegenemyers/MERQURY.FK
MBG	-	https://github.com/maickrau/MBG
PretextView	0.2	https://github.com/wtsi-hpag/PretextView
purge_dups	1.2.3	https://github.com/dfguan/purge_dups
SALSA	2.2	https://github.com/salsa-rs/salsa

### Wellcome Sanger Institute – Legal and Governance

The materials that have contributed to this genome note have been supplied by a Darwin Tree of Life Partner. The submission of materials by a Darwin Tree of Life Partner is subject to the **‘Darwin Tree of Life Project Sampling Code of Practice’**, which can be found in full on the Darwin Tree of Life website here. By agreeing with and signing up to the Sampling Code of Practice, the Darwin Tree of Life Partner agrees they will meet the legal and ethical requirements and standards set out within this document in respect of all samples acquired for, and supplied to, the Darwin Tree of Life Project.

Further, the Wellcome Sanger Institute employs a process whereby due diligence is carried out proportionate to the nature of the materials themselves, and the circumstances under which they have been/are to be collected and provided for use. The purpose of this is to address and mitigate any potential legal and/or ethical implications of receipt and use of the materials as part of the research project, and to ensure that in doing so we align with best practice wherever possible. The overarching areas of consideration are:

•   Ethical review of provenance and sourcing of the material

•   Legality of collection, transfer and use (national and international) 

Each transfer of samples is further undertaken according to a Research Collaboration Agreement or Material Transfer Agreement entered into by the Darwin Tree of Life Partner, Genome Research Limited (operating as the Wellcome Sanger Institute), and in some circumstances other Darwin Tree of Life collaborators.

## Data Availability

European Nucleotide Archive: *Ailanthus altissima* (tree of heaven). Accession number PRJEB47393; https://identifiers.org/ena.embl/PRJEB47393 ( Wellcome Sanger Institute, 2022) The genome sequence is released openly for reuse. The *A. altissima* genome sequencing initiative is part of the Darwin Tree of Life (DToL) project. All raw sequence data and the assembly have been deposited in INSDC databases. The genome will be annotated using available RNA-Seq data and presented through the Ensembl pipeline at the European Bioinformatics Institute. Raw data and assembly accession identifiers are reported in Table 1.

## Author information

Members of the Royal Botanic Gardens Kew Genome Acquisition Lab are listed here: https://doi.org/10.5281/zenodo.4786680.

Members of the Plant Genome Sizing collective are listed here: https://doi.org/10.5281/zenodo.7994306.

Members of the Darwin Tree of Life Barcoding collective are listed here: https://doi.org/10.5281/zenodo.4893703.

Members of the Wellcome Sanger Institute Tree of Life programme are listed here: https://doi.org/10.5281/zenodo.4783585.

Members of Wellcome Sanger Institute Scientific Operations: DNA Pipelines collective are listed here: https://doi.org/10.5281/zenodo.4790455.

Members of the Tree of Life Core Informatics collective are listed here: https://doi.org/10.5281/zenodo.5013541.
