# Supplementary material for: The genome sequence of the tree of heaven, Ailanthus altissima (Mill.) Swingle, 1916
Source: Wellcome Open Res. 2023 Jul 25;8:321. [Version 1] doi: 10.12688/wellcomeopenres.19628.1 (PMC10472066; doi:10.12688/wellcomeopenres.19628.1)
Supplement: Peer review history

# Peer review: Reviewer response for version 1

**DOI:** 10.21956/wellcomeopenres.21744.r63916

**Published:** 2023-08-31

**Authors:** Jim Leebens-Mack

**Affiliations:** 1 Department of Plant Biology, 4505 Miller Plant Sciences, University of Georgia, Athens, Georgia, USA

The authors present a reference quality, chromosomal genome assembly for *Ailanthus altissima. *As far as I know, this is the first  chromosomal genome assembly for any member of the family Simaroubaceae (Sapindales) and it will be quite useful for comparative genome analyses across rosids, the eudicots and all flowering plants.

 A very minor point for this report and all Darwin Tree of Life genome reports - scaffold N50 stats are meaningless for chromosomal assemblies made using "3rd generation" sequencing technologies. The N50 stats reflect the chromosomal size distributions rather than the quality of the assembly. Numbers of gaps in the scaffold assemblies are more informative, but again the gap number may say more about the distribution of repeats rather than the quality of the assembly. If the authors chose to keep the N50 statistics in this report, it would be helpful to add a sentence or two on how they should be interpreted.

Are sufficient details of methods and materials provided to allow replication by others?

Yes

Is the rationale for creating the dataset(s) clearly described?

Yes

Are the datasets clearly presented in a useable and accessible format?

Yes

Are the protocols appropriate and is the work technically sound?

Yes

Reviewer Expertise:

Phylogenomics and comparative plant genomics

I confirm that I have read this submission and believe that I have an appropriate level of expertise to confirm that it is of an acceptable scientific standard.

# Peer review: Reviewer response for version 1

**DOI:** 10.21956/wellcomeopenres.21744.r63917

**Published:** 2023-08-16

**Authors:** Xin Liu

**Affiliations:** 1 State Key Laboratory of Agricultural Genomics, BGI (Beijing Genomics Institute)-Shenzhen, Shenzhen, China

The manuscript by Rowan *et al.* described the genome assembly of *Ailanthus altissima*. They applied PacBio, 10X and HiC sequencing to obtain the sequencing data, and they assembled a chromosome-level genome, with good quality. The method used should be appropriate thus the generated dataset should be valuable for future studies. This type of manuscript is quite straightforward, and I think it is OK for publish as data note. I only have several suggestions for the authors to consider.

1. With the cytometric evaluation, the genome size was estimated to be 1,170 Mb, which is larger than the assembled genome. Although there would be some acceptable reasons for this, it would be good to have some more information. Have you estimated the genome size using Kmer analysis? If so, it would be good to know the estimation using the sequencing data.
2. The authors mentioned about the manual curation of the genome assembly to remove some assembly errors. Especially for the haplotypic duplication, it would be better to mention the definition of this type of assembly error. Also, for the contigs corresponding to the second haplotype, it would be more informative to mention the size of the contigs. 

Are sufficient details of methods and materials provided to allow replication by others?

Yes

Is the rationale for creating the dataset(s) clearly described?

Yes

Are the datasets clearly presented in a useable and accessible format?

Yes

Are the protocols appropriate and is the work technically sound?

Yes

Reviewer Expertise:

Plant genomics

I confirm that I have read this submission and believe that I have an appropriate level of expertise to confirm that it is of an acceptable scientific standard.
